# Development of a Video-Test of Emotional Intelligence for Teachers (ViTIED)

**DOI:** 10.3390/jintelligence13010003

**Published:** 2024-12-30

**Authors:** María-Pilar Berrios-Martos, Raquel Palomera

**Affiliations:** 1 Department of Psychology, University of Jaén, Campus Las Lagunillas s/n, 23071 Jaén, Spain; 2 Department of Developmental and Educational Psychology, Universidad de Cantabria, Av. de los Castros, s/n, 39005 Santander, Spain; palomerr@unican.es

**Keywords:** emotional intelligence, teacher, secondary education, test

## Abstract

Emotional Intelligence (EI) in teaching is associated with various educational outcomes and processes. However, it has typically been measured through self-reports and general EI assessments, lacking a specific performance test with greater ecological validity in relation to the demands of the professional educational context. This study describes the development and validation results of the Video-Test of Emotional Intelligence for Teachers (ViTIED), a new performance-based measure to assess the EI of secondary education teachers based on ability EI model and the situational judgment test paradigm. The test comprises 12 video scenes designed to elicit intra- and interpersonal processes, as well as both positive and negative emotions. A total of 163 Spanish teachers (36% male, 64% female; mean age = 40.32 years) completed the ViTIED, along with personality, perceived EI, and burnout assessments. Test scores provide initial evidence of adequate reliability, as well as content, convergent, and divergent validity. Continued validation of this measure will benefit evaluation and intervention processes with teachers, as well as research on the impact of teachers’ EI on the teaching–learning processes and the well-being of the educational community.

## 1. Introduction

Teaching is an emotional labor considered a high-risk profession due to the high impact of several risk factors on educators’ daily lives. Teachers are required to cope with a wide variety of stressors, including workload, role ambiguity, lack of workplace social support, and classroom management difficulties, among others (Mérida-López and Extremera 2017). In fact, conflict is a large part of the interpersonal relationships in a school setting, which are essential in the teaching profession, and the presence of conflict often affects the quality of teaching, as well as the relationships within and the well-being of the entire educational community (Rudasill et al. 2018). Therefore, teaching professionals tend to report high levels of occupational stress and burnout. According to Maslach and Jackson (1986), burnout is characterized by three symptoms: emotional exhaustion, conceived as feeling physically and emotionally overextended; depersonalization, defined as a distant attitude toward students; and a loss of self-confidence and lack of personal accomplishment. This syndrome has been extensively studied in teaching populations, consistently showing a negative impact on the quality of teaching and learning processes, the school climate, and relationships, as well as the teacher’s health and well-being (Agyapong et al. 2022).

Looking for protective factors against these issues, studies of personal resources such as emotional intelligence have proliferated in educational research.

According to the Ability Model, EI is defined as “the capacity to reason about emotions and use emotional knowledge to enhance and guide thought and behavior, enabling better decision-making, solving social problems, and effectively adapting to the environment” (Mayer et al. 2008, p. 511).

Research indicates that teachers with higher EI report lower levels of burnout (Kotaman et al. 2022; Mérida-López and Extremera 2017; Sánchez-Pujalte et al. 2023) use more positive and adaptive coping strategies when facing stress (Pulido-Martos et al. 2022; Valente and Lourenço 2020), experience greater engagement and job satisfaction (Mérida-López et al. 2022; Wang 2022), and demonstrate higher levels of empathy and well-being (Mérida-López et al. 2022; Porras-Carmona et al. 2020). Moreover, teachers’ EI fosters the development of students’ socioemotional skills (Berrios-Martos et al. 2016; Jennings and Greenberg 2009), along with their psychological adjustment, engagement, and academic performance (Wang 2022; Welmilla 2020).

However, most research in this field is conducted via self-reports or tests assessing general EI. To advance research on the role of teachers’ EI in educational processes and support their training and assessment, assessment tools are needed that provide reliable and valid results for evaluating EI as it applies to resolving everyday conflict situations involving members of the educational community. In the educational context, the most effective emotional strategies may vary depending on the situation the teacher faces (Rodríguez-Pérez et al. 2021). Therefore, it is essential to define the specific and concrete job situations where a set of competencies must be applied to resolve them successfully.

## 2. Evaluation of Emotional Intelligence: Advances and Challenges

There are currently two main co-existing theoretical approaches to the EI construct: (1) The Ability Model (Mayer and Salovey 1997) conceptualizes EI as the ability to process emotional information, which comprises the following four hierarchically intercorrelated branches, all of which must be assessed using performance tests: (1) perceiving, (2) using, (3) understanding, and (4) managing emotions. Perceiving and using emotions constitute the Experiential EI Area (ability to perceive, respond, and manipulate emotional information without necessarily understanding it), while the understanding and managing of emotions constitute the Strategic EI Area. To manage emotions adequately, one must be able to distinguish between emotions and label them accurately, as well as select and deploy strategies to alter them. The two latter EI branches are strategic in the sense that they provide the basis for charting an emotional course for oneself and others according to personal and social needs. Together, these two areas form a general EI factor. In contrast, the (2) Trait Model (e.g., Petrides and Furnham 2000) conceptualizes EI as a constellation of emotion-related characteristics that must be assessed using self-report questionnaires. Finally, (3) mixed EI models define EI as a combination of personality traits and other characteristics (self-esteem, achievement orientation, and stress tolerance, among others) (Bar-On 2006; Goleman 1998), also assessed through self-report measures.

Currently, a series of reliable and valid measures are available based on different approaches: (1) Performance-based EI, assessed as a type of intelligence similar to cognitive intelligence, using performance tests. Studies focusing on performance-based EI are scarce, and most have used the Mayer, Salovey, and Caruso Emotional Intelligence Test (MSCEIT; Mayer et al. 2002). This test uses two scoring methods: consensus population and experts. Although the correlation between the expert method and the consensus method is high, it has been proven that experts are more reliable judges and converge on correct answers when the research, as in this case, has established clear criteria for responses. Thus, the expert criterion may be preferable to general consensus (Mayer et al. 2003). The expert method can be used for scoring, where an individual’s ability is compared with the consensus of EI experts, with each possible response weighted by the proportion of experts who selected that response. While this performance measure avoids the biases inherent in subjective assessments, it also has several limitations (Extremera and Fernández-Berrocal 2004; Fernández-Berrocal and Extremera 2005): (a) it is lengthy and expensive; (b) it evaluates emotional strategies only after negative events have occurred, without assessing strategies that could prevent such events; (c) it presents static photographs, failing to capture the dynamism of micro-expressions at real-life speed; (d) it assesses each branch of EI separately through different tasks and targets, rather than jointly; and (e) it lacks context-specific situations, reducing its ecological validity. (2) Self-report instruments, which measure the perception of one’s own emotional abilities. Self-report measures are less expensive, easier to administer, and take less time than performance tests. However, responses can be biased by social desirability, difficulties in meta-knowledge, or comparison bias. Self-report measures are also weakly related to performance-based measures and show low discriminant validity compared with personality measures (Brackett and Geher 2006; Brackett and Mayer 2003). A key methodological challenge in avoiding mono-method bias (Matthews et al. 2007) is the need to design additional instruments that can overcome these limitations. To address this, measures based on the situational judgment test (SJT) paradigm have been developed. The SJT evaluation paradigm is based on the premise that the decisions and actions chosen in the presented situations are indicative of an individual’s actual performance and abilities, systematically and intentionally measuring procedural knowledge, not just declarative knowledge (Lievens and Motowidlo 2016). Furthermore, it has been proven that in the educational context, the most effective socioemotional strategies can vary depending on the problem and/or situation faced by the teacher (Rodríguez-Pérez et al. 2021), who must have an “executive functioning” in which they can optimize their cognitive processes to guide them towards the resolution of complex situations (Tirapu-Ustarroz et al. 2002). These executive functions require the ability to set goals and objectives, plan strategies to achieve them, and carry out effective actions (García-Molina et al. 2007). Therefore, it is necessary to define the concrete and specific situations of the job in which a series of competencies will have to be applied to resolve them successfully, thus increasing the ecological, apparent, and incremental validity to predict performance in high-psychosocial-risk work environments (Lievens and Chan 2017; Whetzel and McDaniel 2009), such as teaching. Therefore, in order to assess teachers’ ability to analyze complex emotional situations inherent to their work, understand the implications of different actions, and make appropriate decisions based on the circumstances presented, the paradigm of situational judgment tests can be applied. This assessment paradigm is based on the idea that the decisions and actions chosen in the situations presented are indicative of the individual’s real performance and skills in everyday situations in their work. Therefore, it is considered a valid and reliable way to assess and predict performance in specific contexts, in this case, in education (Lievens and Sackett 2012; McDaniel et al. 2007). In recent years, situational judgment tests have made significant advances in assessment practices for the following reasons: (a) they predict job performance because they measure procedural knowledge about how to behave effectively in various work situations, and (b) this knowledge is not acquired from specific work experience, but rather reflects effects of fundamental socialization processes and personal dispositions and can predict performance in work situations. Therefore, it is desirable to develop situational judgment tests to measure procedural knowledge in a purposeful and systematic manner (Lievens and Motowidlo 2016).

This approach increases ecological, face, and incremental validity in predicting performance in high-psychosocial-risk environments, such as teaching (Lievens and Chan 2017; Whetzel and McDaniel 2009). In the field of EI, and under this paradigm, several instruments have been developed for adult samples, such as the Situational Test of Emotional Understanding (STEU) and the Situational Test of Emotion Management (STEM) by MacCann and Roberts (2008); the Strategic Test of Emotional Intelligence (STEI; Fernández-Berrocal et al. 2019); and the Test of Regulation in and Understanding of Social Situations in Teaching (TRUST, Aldrup et al. 2020; Spanish validation by Martín-Antón et al. 2024). Although all of them have shown good reliability and validation, the first two are not validated in Spain, the second only measures the Strategic Area of EI, and the third, while specific to primary and secondary school teachers, only evaluates emotional regulation and relationship management and does not use dynamic audiovisual media. Furthermore, the use of audiovisual media to assess competencies or skills is a useful, effective, and motivating resource (Bernabé-Valero and Blasco 2015), especially when knowledge instructions are used (i.e., how effective is each of the following strategies for…?), a Likert-type scale is presented for each strategy, and a group of experts is used to carry out the test individually in order to establish appropriate response keys by consensus (Whetzel and McDaniel 2009). In fact, recent studies confirm the advantages of video-based STJs applied in the educational context. Among them are the potential to reduce subgroup differences, the opportunity for applicants to judge the interpersonal cues that are presented in video formats, and more favorable applicant reactions (i.e., novice teachers in training) than those using text because they consider them more attractive (Bardach et al. 2020), and they predict decision making in classroom management (Weyers et al. 2024).

According to this, the ViTIED presents a series of advantages that overcome the limitations of the previous EI instruments, the most prominent of which are described here. (1) Extension, application time, and cost: The ViTIED is an execution test that evaluates the four branches of the Mayer and Salovey (1997) model through a simple and coherent structure, and its application is agile and relatively fast, consisting of 12 mini-videos of approximately 1 min each, with an estimated total completion time of the test of 25 min, compared with the 45 required to answer the 141 items of the MSCEIT. On the other hand, it is a free audiovisual test under a Creative Commons CC BY-NC-ND license that can be carried out using new technologies, while the MSCEIT requires a considerable expense both in its online and paper versions. (2) Novel and realistic format: the videos presented are more credible and similar to reality, and in this case, more specific to the teacher than a static situation, written and drafted by IE experts who have previously summarized and selected the most relevant information of the case, as occurs in the MSCEIT. (3) Comprehensive assessment: Unlike the MSCEIT, which evaluates each branch separately, the audiovisual test that we propose evaluates the four branches jointly and in an integrated way, replicating how we cognitively process emotional information. To respond to this test, a series of learned emotional skills must be put into action and demonstrated as if it were a real-life case. (4) Specificity: the ViTIED presents different real and representative situations of the academic context in which the main obstacles and facilitators that teachers usually face in their educational work appear.

In short, with the ViTIED, we evaluate EI performance by presenting short videos that represent educational situations (six intrapersonal and six interpersonal, depending on whether emotional regulation is applied to oneself or to others) with the main obstacles and facilitators perceived by teachers, according to Martínez and Salanova (2004), habitually experienced in their relationship with the main agents of interaction in the educational community: students, teachers, and students’ families.

## 3. Video-Test of Emotional Intelligence for Teachers (ViTIED)

To further this goal, the Video-Test of Emotional Intelligence for Teachers (ViTIED) was designed. It assesses performance-based EI using the Ability Model of EI (Mayer and Salovey 1997) through the SJT paradigm and employs the expert method for scoring. Conducted online, the test has a simple and coherent structure, making it efficient and relatively quick to administer. It consists of 12 short videos, each lasting approximately 1 min, with an estimated total completion time of 25 min. Moreover, it is a free audiovisual test licensed under a Creative Commons CC BY-NC-ND license.

For the development of the test, the teaching situations were first selected by considering common educational challenges as main barriers and facilitators. They were identified by teachers in their daily work with the main actors in the educational community: students, teachers (including the administration of the school system), and students’ families in the study conducted by Salanova et al. (2005). The obstacles include the following: (1) a lack of interest and motivation to learn, (2) inappropriate student behavior (disrespect toward teachers and/or classmates), (3) negative attitudes of parents regarding their children’s learning, (4) difficulties in coordination among teachers and/or the school administration, (5) difficulty in accessing school resources, (6) teachers’ low perception of self-efficacy, and (7) students’ family problems. The facilitators include the following: (1) student learning and progress, (2) active cooperation and participation of students, (3) establishing positive emotional bonds with students, (4) mutual support among teachers when needed, and (5) enjoyable moments with students.

Secondly, the videos representing the selected situations were chosen. According to Article 32 of Royal Legislative Decree 1/1996, of April 12, which approves the revised text of the Intellectual Property Law in Spain, it is permissible to use brief excerpts from films or series for educational, non-commercial purposes. To select the short videos for the test, the series *Merlí*, produced by Veranda TV, created by Héctor Lozano, and directed by Eduard Cortés, was chosen. The series portrays the daily life of a high school teacher who faces challenging situations with adolescents, families, and other faculty members. Two EI experts independently watched the series to identify potential scenes that represent the situations outlined in the first step. They then reached a consensus on the scenes to be selected based on their representativeness and clarity in line with the objectives of the scene.

To analyze content validity, 14 secondary education teachers viewed the 12 selected videos and answered the following questions for each: “What situation do you think the scene you just watched represents?” (12 response options based on the previously identified obstacles and facilitators, with only one option allowed) and “To what extent do you consider this scene representative of the teaching profession?” (five-point Likert scale).

Subsequently, to construct the items for each branch of EI, participants were asked to answer several open-ended questions: “What emotions is character A expressing in this situation?” “What do you think they are thinking that makes them feel this way?” “What would be the best strategies to help B (teacher/student/parent, depending on the case) manage the situation and/or feel better?” Based on the frequency and diversity of the responses, and considering different theories on emotions and emotional regulation strategies (e.g., Mayer and Salovey 1997; Niven et al. 2009), the items were drafted in Google Forms.

The resulting test consists of 12 situations—six intrapersonal and six interpersonal—each of which measures the four branches of EI through a total of 48 questions (228 items). Of these, 36 questions provide five response options, and 12 (related to emotional facilitation) provide four options; each option is answered on a five-point Likert scale. For example, Video 1, selected from episode 4 of season 2 of the series, lasts 35 s and portrays a scene of apathy and disinterest from a student (Berta) who cannot answer the teacher’s question. The questions for this video are as follows:

1. (Interpersonal emotional perception) To what extent do you think Berta is displaying each of the following emotions? (1 = not at all, 5 = very much): (a) frustration, (b) apathy, (c) fear, (d) shame, (e) contempt.
2. (Emotional facilitation) To what extent will feeling this way help Berta to: (1 = not at all, 5 = very much): (a) pay attention for the rest of the class, (b) complete her homework for the next day, (c) help a classmate, (d) analyze the possible causes and solutions for what just happened.
3. (Emotional understanding) What might Berta be thinking to feel this way? (1 = not at all, 5 = very much): (a) “I don’t care about anything”, (b) “I’m making a fool of myself”, (c) “Knowing that fact is useless to me”, (d) “Today is just not my day”, (e) “The teacher has it in for me”.
4. (Interpersonal emotional regulation) What can the teacher do to help Berta feel better? (1 = very ineffective, 5 = very effective): (a) downplay the issue as an occasional mistake, (b) insist that she must fulfill her student responsibilities, (c) ignore the mistake and ask another student, (d) encourage her to try harder, (e) offer academic support to help with her studies.

The first version of the test was evaluated by ten EI experts from various Spanish universities. After being informed about the test and its context of use, they independently provided feedback on the content of the items, following the recommendations of Escobar-Pérez and Cuervo-Martínez (2008). First, they rated the degree to which the items in each scenario were (1) clear (the item is easily understood due to the suitability of its structure and content for the target population), (2) coherent (the item is logically related to the dimension it measures according to the model), (3) relevant (the item provides relevant information about a necessary aspect of the dimension being measured, so it should be retained), and (4) sufficient (it contains the necessary number of items per dimension for its complete evaluation), using a five-point Likert scale. Then, through an open-ended question, they could comment on whether any item or response option needed to be added, modified, or eliminated, and, if necessary, suggest a better alternative.

After incorporating the suggestions from the content experts, the final version of the test was developed. This version was completed by 13 additional EI experts from various Spanish universities, and their scores were used to establish the criteria for expert scoring. Finally, the test was administered to a sample of secondary education teachers to analyze the validity of the results.

The aim of this study is to present the design and preliminary analysis of the ViTIED test for secondary education teachers.

## 4. Materials and Methods

Several analyses have been carried out to validate the instrument. This includes analyzing content validity, as well as the reliability of the obtained scores, and interpreting concurrent validity in relation to perceived EI scores, discriminant validity compared with personality scores, and criterion validity in relation to burnout scores. Additionally, we examine the relationship between the test results, age, and years of professional experience.

### 4.1. Data Collection

After obtaining informed consent, the assessment instruments were completed online, voluntarily and anonymously, in approximately 40 min. Teachers received the invitation via their institutional email.

#### 4.1.1. Participants

The sample of secondary education teachers used to validate the content of the audiovisual scenes—selected as representative of the teaching profession and its various obstacles and facilitators—consisted of 14 professionals (64.3% women, mean age = 47.8 years, SD = 7.6), with an average of 18.9 years (SD = 9.8) of teaching experience.

Ten EI experts validated the content (60% women, mean age = 45.4 years, SD = 7.5), with an average of 18 years (SD = 8.4) of teaching experience and 13.62 years (SD = 5.8) of research experience. Seventy-five percent were familiar with the series *Merlí*.

The sample of experts used to obtain consensus scoring consisted of 13 Spanish researchers (50% men, 50% women, mean age = 42.37 years, SD = 9.9), with an average of 16.56 years (SD = 10.6) of teaching experience and 16.43 years (SD = 9.4) of research experience in the field of EI. Fifty percent were familiar with the series *Merlí*.

The convenience sample used for construct validation consisted of 163 secondary education teachers (64% women), with a mean age of 40.32 years (SD = 11.39), from various regions of Spain (69.9% from Andalusia, 25.3% from Cantabria, 1.2% from Aragón, and 3.6% from Madrid). They worked in private (11%), public (74.4%), and semi-private (14.6%) schools and had an average of 11.46 years (SD = 10.75) of teaching experience. The teachers worked at various levels, including compulsory secondary education (35.3%), sometimes combined with high school teaching (23.8%) or vocational training (7.3%), while one-third of the sample (33.6%) taught across all these levels. The majority (67.7%) were unfamiliar with the series *Merlí*.

#### 4.1.2. Instruments

*Sociodemographic data.* Information was collected on gender, age, region, educational stage, type of school, years of teaching experience, and familiarity with the series *Merlí*.

*Video-Test of Emotional Intelligence for Teachers* (ViTIED). This test consists of 12 school situations (228 items), represented through short videos depicting the main obstacles and facilitators perceived by teachers. In each scene, teachers must complete four tasks that assess the four branches of the Ability Model of EI, using five-point Likert scales.

*Spanish transparent version of Goldberg’s Big Five 50 Personality Markers* (Goldberg 1992; shortened Spanish version by García et al. 2004). It is composed of five scales, each with five items: Extraversion, Agreeableness, Conscientiousness, Neuroticism, and Intellect. The bipolar scale for each pair of adjectives ranges from 1 (very characteristic of trait A) to 5 (neither trait A nor trait B) to 9 (very characteristic of trait B).

*Workgroup Emotional Intelligence Profile—Short Version* (WEIP-S) (Jordan and Lawrence 2009; Spanish adaptation by Lopez-Zafra et al. 2012). The scale consists of 16 items, rated on five-point Likert scales (1 = strongly disagree; 5 = strongly agree), that evaluate four dimensions: Awareness of Own Emotions (e.g., “I can explain my emotions to other team members”), Regulation of Own Emotions (e.g., “When I am frustrated with a team member, I can overcome my frustration”), Awareness of Others’ Emotions (e.g., “I notice their true feelings even if they try to hide them”), and Regulation of Others’ Emotions (e.g., “I can cheer up team members when they feel down”). Participants were asked to complete the scale three times, thinking about each of the following groups with which they interact most in their daily work: students, other teachers, and students’ families. The mean score for each group was used to calculate each dimension.

*Maslach Burnout Inventory—General Survey* (MBI-GS) (Maslach et al. 1996; Spanish adaptation by Salanova and Schaufeli 2000). This instrument consists of 16 items that assess three dimensions: emotional exhaustion (e.g., “I feel emotionally drained from my work”), cynicism (e.g., “I have become less enthusiastic about my work”), and professional efficacy (e.g., “I can effectively solve the problems that arise in my work”). All items are rated on a seven-point Likert scale, reflecting the frequency of the experience.

#### 4.1.3. Compliance with Ethical Standards

The study was approved by the Human Research Ethics Committee of the University of Jaén and conducted in accordance with the Declaration of Helsinki (2013).

#### 4.1.4. Data Analysis

SPSS v.22.0 (IBM, Chicago, IL, USA) was used to calculate Fleiss’ Kappa and Kendall’s *W* statistics for interrater agreement, descriptive statistics, correlation analyses, internal consistency, and Student’s *t*-tests. AMOS v.16 (IBM, Chicago, IL, USA) was used to perform Confirmatory Factor Analysis (CFA). All skewness and kurtosis values were within the acceptable range of ±2 (Gravetter and Wallnau 2014). Additionally, Mardia’s multivariate coefficient showed a value of −1.39, below the cut-off of 5.00 suggested by Bentler and Wu (2005). The results of the sphericity test (*p* < 0.05) and the Kaiser–Meyer–Olkin (KMO = 0.85) measure of sampling adequacy indicated that the use of CFA was appropriate. These statistics confirmed normality, so the CFA was conducted using the Maximum Likelihood method.

Following the recommendations of Schreider et al. (2006), additional model fit indices were used, including absolute fit measured by Chi-square (χ^2^), with significance values (*p* > 0.05); the Comparative Fit Index (CFI ≥ 0.90); the Tucker–Lewis Index (TLI ≥ 0.90); the Normed Fit Index (NFI ≥ 0.90); the Root Mean Square Residual (RMR, close to zero); the Root Mean Square Error of Approximation (RMSEA ≤ 0.05); and the Akaike Information Criterion (AIC).

Two models derived from the Ability Model of EI were analyzed. In both cases, variables were observed through tasks, taking into account previous studies on the factor structure of EI ability measures (Rivers et al. 2012). One model consisted of a general factor model, with a higher-order latent factor grouping the four tasks into a single general factor. The other model established two areas, treating the experiential and strategic domains as higher-order latent factors and grouping the tasks into two related first-order latent factors. There were no missing data, as responses were required in the online test format.

## 5. Results

### 5.1. Content Validity

The level of agreement among teachers regarding the classification of the type of obstacle or facilitator represented in the scenes was significant, with *K* = 0.22, *p* < .01. There was also a significant positive association concerning the perceived representativeness of the teaching profession in the audiovisual scenes, with a concordance coefficient of *W* = 0.30, *p* < .01. The level of agreement among expert judges was significant for the content of the following items: clarity (*W* = 0.50), coherence (*W* = 0.56), relevance (*W* = 0.49), and sufficiency (*W* = 0.47), all with *p* < .001.

The reliability among the second group of experts when completing the test resulted in an intraclass correlation coefficient of 0.97.

### 5.2. Factor Structure

**Figure 1:**
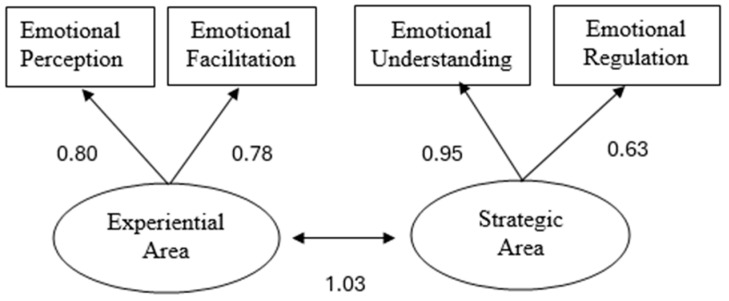
Model with two areas of EI.

The fit parameters for the general factor model and the areas model are presented in Table 1. The one-factor model did not show an adequate goodness-of-fit, whereas the two-area model yielded appropriate fit indices. Figure 1 presents the two-area model along with the standardized beta coefficients.

### 5.3. Descriptive Statistics, Reliability, and Correlations Among Subscales

Table 2 presents the descriptive statistics and reliability for the subscale scores of the test (Cronbach’s alpha, McDonald’s omega). Cronbach’s alpha coefficients for the different branches ranged from 0.89 to 0.94. The alpha coefficients for the experiential and strategic areas were 0.93 and 0.96, respectively. Omega coefficients for the branch scores also showed a high range (0.85–0.94), with high area scores of 0.95 and 0.92.

Independent sample *t*-tests were conducted, and the results are presented in Table 2. No significant differences were found between teachers who were familiar with the television series (from which the video clips in the test were taken) and those who were not.

Correlations among the ViTIED subscales were examined (Table 3), revealing significant positive associations ranging from moderate to high, including the association between the two areas. Due to the potential for multicollinearity among the factors, the Variance Inflation Factor (VIF) was analyzed (see Table 3), with all values below 10 (Kutner et al. 2004).

### 5.4. Correlations with Years of Experience and Age

Pearson correlations revealed low, non-significant positive associations between the ViTIED subscales and both years of teaching experience and age, except for a low but significant positive correlation between age and both emotional perception and the Experiential Area (Table 3).

### 5.5. Associations with Personality Traits, Perceived Emotional Intelligence in Teams, and Burnout

As shown in Table 4, all the subscale scores for the criterion variables demonstrated adequate reliability. The correlations between the ViTIED subscales and personality traits were not significant, except for low but significant positive correlations between emotional facilitation and conscientiousness and between emotional regulation and intellect. Regarding the relationship between the ViTIED subscales and perceived EI (both personal and interpersonal emotional understanding and regulation), all correlations were positive and significant, particularly with the branches of emotional understanding and regulation, as well as with the two areas and Total EI.

Finally, for emotional exhaustion, low but significant negative correlations were found with emotional perception and understanding, as well as with the two EI areas and total score. Cynicism showed significant negative associations with all branches, areas, and the total EI score. Lastly, self-efficacy was significantly, positively, and moderately correlated with all EI scores, with a particularly strong correlation with emotional regulation.

## 6. Discussion and Conclusions

In this study, we presented the development of a situational judgment test (SJT) to measure the Emotional Intelligence (EI) of secondary education teachers as an ability using a video- test and presented results providing evidence of the measure’s validity and reliability.

The results indicate adequate face validity based on teachers’ judgments regarding the representativeness of the presented circumstances, as well as strong evidence of content validity according to evaluations by EI experts.

The results also support the two-area model of EI (Strategic and Experiential), showing high reliability indices, similar to the TIEFBA test, which measures EI in adolescents using the SJT paradigm and also found the two-area model to be a better fit (Fernández-Berrocal et al. 2018). Additionally, we found high, significant positive correlations among the different subscales of the ViTIED. This structure is empirically and theoretically justified by the Ability Model of EI (Mayer et al. 2012). Therefore, the results of the test demonstrate evidence of validity based on the instrument’s internal structure. The Ability Model of EI suggests that the Experiential EI area represents the direct processing of information in the immediate environment, unmediated by high-level strategic planning. In contrast, the Strategic EI area is thought to encompass strategic judgments and high-level deliberate processing of emotional information (MacCann et al. 2014). Looking at the results within the Blömeke et al. (2015) model of professional competence, where competence is viewed as a continuum of dispositions related to situation-specific skills that are expressed in an observable behavior (performance), the ViTIED areas would provide information about the three situation-specific skills (the experimental area more related with perception and the strategic area with interpretation and decision-making), which refer to the cognitive processes in specific work situations and are conceptualized as a link between disposition and performance. Derived from this, to assess all EI dimensions and potentials, we should use different assessment methods in research and practice; for example, to assess EI as a dispositional trait, we should use self-report instruments, and to measure EI as an ability for specific skills, out of a specific context, we could use general EI instruments. Contextual observation tools and qualitative methods will also help to understand teachers’ EI processes and roles in educational settings.

Moreover, ViTIED scores provide evidence of validity in relation to other variables. First, discriminant validity is supported by the low associations with two personality traits (conscientiousness and intellect), which aligns with previous literature. According to Ashton et al. (2000), EI tests should relate to personality traits in the same way as other intelligence tests, with correlations of *r* = 0.30 or less. Specifically, studies using SJTs such as the STEU, STEM, and TRUST have found low to moderate correlations with traits like openness to experience, agreeableness, and conscientiousness (Aldrup et al. 2020; Libbrecht and Lievens 2012; MacCann and Roberts 2008). Future research will aim to replicate these findings with ViTIED, evaluating personality traits using other measures based on the “Big Five” model. These personality traits are also part of teacher dispositions, affecting their competence.

Second, in terms of concurrent validity, the scores on the ViTIED subscales correlate positively and significantly, though moderately, with perceived EI (emotional understanding and regulation of both self and others). This suggests that teachers’ perceived EI, as a dispositional trait, is linked to the development of emotional abilities in the context of their interactions with various members of the educational community.

Third, the test scores show significant associations with the criterion variable of teacher burnout: negative correlations with emotional exhaustion and cynicism and moderate to high positive correlations with professional self-efficacy, particularly emotional regulation. This suggests that emotional regulation may play a crucial role in fostering beliefs about one’s ability to plan, organize, and implement actions to achieve goals, as described by social learning theory (Bandura 2001). These beliefs increase motivation to complete tasks and persist in them despite challenges. These findings align with other studies that have also found strong correlations between emotional regulation, self-efficacy, and burnout in teachers (e.g., Brackett et al. 2010; Fathi and Derakhshan 2019; Geraci et al. 2023; Sánchez-Pujalte et al. 2023). Similarly, in other professions that require a high level of emotional resilience, such as medicine and nursing, the findings are comparable (e.g., Horne et al. 2024; Yu et al. 2023). In light of the above results, it is plausible that the same methodology used in the development of ViTIED could be used to design a similar assessment tool for application in other high-stress occupational categories.

Regarding age, the results show low, non-significant positive associations with the ViTIED subscales, except for emotional perception, which shows a slight positive relationship. The Ability Model (Mayer and Salovey 1997) suggests that EI is a genuine form of intelligence that increases with age and experience (Extremera et al. 2006; Kafetsios 2004), although this relationship is not linear but rather follows an inverted U shape (Cabello et al. 2016). Since all participants in our study were adults, we expected to find little relationship between EI and teachers’ age. In the same line, years of professional experience did not show a relationship with teacher EI in task resolution, underscoring both the utility of EI and the need for training, regardless of whether teachers are fresh or experienced. Evidence-based teacher preparation programs are especially needed to develop pre-service teachers’ social and emotional learning to foster well-being (Corcoran and O’Flaherty 2022).

Our findings show promising results for the ViTIED, which appears to be an innovative instrument for assessing teacher EI. There is adequate initial evidence of construct validity, as well as the expected criteria for tests measuring EI (MacCann et al. 2014; Mayer et al. 2008; Mayer et al. 2012): (1) it has a clear internal structure that mirrors the theoretical framework of the Ability Model of EI (Mayer and Salovey 1997), with adequate reliability indices; (2) it is distinct from personality traits; and (3) it shows moderate, positive correlations with perceived EI, as assessed by self-report measures. Correlations between SJTs (e.g., TRUST, STEM, and TEMT) and self-report measures (e.g., TEIQue and SREIS) are typically moderate (Neubauer and Hofer 2022; Martín-Antón et al. 2024; Sharma et al. 2013), suggesting that these instruments assess different yet related psychological processes (emotional self-efficacy vs. reasoning about emotions) (Brown et al. 2023). In our case, these correlations may be due to both instruments aligning with the realities of the teaching profession.

However, our study has some limitations that require caution in interpreting the preliminary results presented, particularly in terms of sample representativeness and cultural bias, which may affect the generalizability of the findings. For example, although the sample reflects the usual proportions of men and women in the teaching profession, a larger sample size with more men would be needed to develop gender validation analyses. Further, although the video-based SJTs = paradigm has several advantages compared with text formats (e.g., reduction in gender effects), some limitations still appear related to ethnicity effects (Bardach et al. 2020). In this sense, some geographical regions are underrepresented in the sample, which may limit the generalizability and validity of the results. Although it is expected that similar results will be found in the rest of the Spanish regions, since they share the same educational system, future research is needed to extend the sample to more territories and ensure its generalizability and validity. Moreover, even though no significant differences were found between teachers who were familiar with the television series (from which the video clips in the test were taken) and those who were not, the test could be adapted for educational contexts with less cultural familiarity with the series, different education system or language. In order to validate the test to be used with other cultures and languages, it would be necessary to subtitle the videos and items using a back translation method. A pilot study would also need to be carried out to see whether teachers identify the same pedagogical challenges, in terms of barriers and facilitators, in their day-to-day professional experiences as they do in the videos, again based on the classification proposed by Salanova et al. (2005). In some cases, it will be necessary to select or create different videos adapted to the culture and daily challenges of the teachers and to adapt or obtain new response items from the teachers. This process would follow the same detailed construction steps as described above.

The fact that the test is an online video-test facilitates data collection and data entry in the research but poses potential challenges on a large scale, as the use of the test requires a technical infrastructure: internet connection, computer device (mobile or PC), headphones, or audio speakers. Although the test provides precise instructions to facilitate the autonomy of the person being assessed, it would also be advisable to instruct the evaluators to avoid bias, e.g., by not inducing answers through information or clarification, but also to be able to reliably manage the correction and interpretation of scores with statistical software skills.

Therefore, future research replicating the analyses with a larger and more heterogeneous sample will help us to confirm these findings and extend the evidence for validity and reliability. It would also allow us to obtain consensus scores for test correction. We recommend the development of longitudinal studies to examine whether ViTIED scores predict long-term improvements in teaching performance and student outcomes. For example, what are the effects of teacher EI on various critical educational issues such as school climate, teaching and learning processes, student academic and social performance, engagement, and well-being? In this line, it will help to assess whether a teacher’s EI has any effect on teacher or student behavior and to analyze the contribution of each EI branch or area to the accurate prediction of such behavior. On the other hand, it would also allow us to examine the underlying processes through which EI positively influences social relationships in school (e.g., self-efficacy beliefs, empathy, managing the emotions of others) and whether teachers with higher EI choose more appropriate coping strategies to resolve situations with students, parents, and colleagues. Future studies could also examine convergent validity with existing measures of general EI skills (e.g., MSCEIT, STEI), with tests of teacher’s classroom management skills (CME-DEcide, Weyers et al. 2024), and use a test–retest design to examine whether scores remain stable over time. In addition, through a quasi-experimental design, the instrument would help to test the effectiveness of EI training programs in increasing teacher’s EI and well-being. Finally, future research would help to gather evidence on this test’s utility and potential adverse effects. This could be achieved through qualitative and/or quantitative data collection methods (Sireci and Benítez 2023).

ViTIED would also be useful in educational practice. For example, feedback from the test could guide the design of specific training programs for initial and in-service teachers, helping to identify the skills or types of conflict that need to be addressed in depth. Also, it could be used in workshops, where test responses are analyzed as a basis for developing emotional management strategies. The tool could be used as a screening method, for example, in recruitment processes, as teacher non-cognitive attributes have been found to be significantly related to teacher effectiveness, more so than traditional knowledge tests (Klassen et al. 2018). It could also be used by in-service teachers as a feedback or self-assessment tool for professional development programs.

It is therefore expected that the use of this test will have a positive impact on school climate, pupils’ academic performance and adjustment, and teachers’ own health, thereby improving the quality of education and well-being in the context of the whole school.

## Figures and Tables

**Table 1:** Fit parameters for one- and two-factor models.

	General Factor	Two-Area
χ^2^ (df)	6.52(2)	1.086(1)
*p*	0.01	0.30
RMR	0.00	0.00
RMSEA	0.89	0.02
CFI	1.00	1.00
TLI	0.00	0.99
NFI	1.00	0.99
AIC	6.00	19.089

**Table 2:** Descriptive statistics and reliability coefficients of the ViTIED.

Subscale	*M* (*DT*)	Alpha	Omega	*t*	*p*
	Total			TV Series	
Emotional Perception	0.31 (0.06)	0.92	0.79	0.11	0.656
Emotional Facilitation	0.32 (0.06)	0.89	0.80	−0.63	0.184
Emotional Understanding	0.34 (0.09)	0.94	0.90	0.75	0.828
Emotional Regulation	0.32 (0.07)	0.92	0.83	0.94	0.346
Experiential Area	0.32 (0.06)	0.93	0.89	−0.32	0.275
Strategic Area	0.33 (0.07)	0.96	0.92	0.93	0.791

**Table 3:** Correlations between ViTIED subscales and sociodemographics.

	1	2	3	4	5	6
1. Emotional Perception	--					
2. Emotional Facilitation	0.62 **	--				
3. Emotional Understanding	0.79 **	0.76 **	--			
4. Emotional Regulation	0.50 **	0.54 **	0.60 **	--		
5. Experiential Area	0.88 **	0.91 **	0.86 **	0.57 **	--	
6. Strategic Area	0.74 **	0.74 **	0.92 **	0.87 **	0.82 **	--
Years of Experience	0.10	0.14	0.06	0.12	0.13	0.10
Age	0.18 *	0.12	0.08	0.11	0.16 *	0.10
VIF	2.68	2.46	4.18	1.6	3.01	3.01

Note: * *p* < .05; ** *p* < .01. VIF: Variance Inflation Factor.

**Table 4:** Reliability, descriptive statistics, and correlations between criterion variables and ViTIED subscales.

	N	E	I	A	C	CEP	CEO	REP	REO	AG	CI	AE
PE	−0.09	−0.02	−0.03	−0.01	0.05	0.12 *	0.24 **	0.26 **	0.27 **	−0.16 *	−0.31 **	0.41 **
FE	−0.04	0.03	0.06	0.13	0.19 *	0.19 *	0.27 **	0.24 **	0.30 **	−0.14	−0.33 **	0.45 **
CE	−0.09	−0.03	0.04	0.05	0.12	0.18 *	0.31 **	0.27 **	0.37 **	−0.16 *	−0.30 **	0.49 **
RE	−0.01	0.02	0.17 *	0.15	0.13	0.31 **	0.24 **	0.28 **	0.46 **	−0.14	−0.29 **	0.72 **
AEX	−0.07	−0.02	0.02	0.07	0.13	0.16 **	0.28 **	0.28 **	0.32 **	−0.17 *	−0.35 **	0.48 **
AES	−0.06	−0.01	0.11	0.11	0.14	0.26 **	0.31 **	0.31 **	0.46 **	−0.17 *	−0.33 **	0.66 **
Alpha	0.84	0.88	0.83	0.74	0.87	0.90	0.93	0.90	0.94	0.87	0.82	0.87
Omega	0.84	0.89	0.81	0.74	0.87	0.89	0.93	0.90	0.94	0.87	0.83	0.88
*M*	6.76	6.99	7.54	7.74	7.61	3.03	3.22	3.38	3.37	2.27	1.8	4.0
*DT*	1.33	1.30	1.05	1.13	1.31	0.78	0.85	0.82	0.92	0.81	0.83	0.68

Note: N, Neuroticism; E, Extraversion; I, Intellect; A, Agreeableness; C, Conscientiousness; CEP, Understanding of Own Emotions; CEO, Understanding of Others’ Emotions; REP, Regulation of Own Emotions; REO, Regulation of Others’ Emotions; AG, Emotional Exhaustion; CI, Cynicism; AE, Self-Efficacy; PE, Emotional Perception; FE, Emotional Facilitation; CE, Emotional Understanding; RE, Emotional Regulation; AEX, Experiential Area; AES, Strategic Area. * *p* < .05; ** *p* < .01.

## Data Availability

Study data are available upon request from correspondence author.
